# Incremental value of cardiovascular magnetic resonance over echocardiography in the detection of acute and chronic myocardial infarction

**DOI:** 10.1186/1532-429X-15-5

**Published:** 2013-01-16

**Authors:** Caroline Jaarsma, Simon Schalla, Emile C Cheriex, Martijn W Smulders, Ivo van Dongen, Patricia J Nelemans, Anton PM Gorgels, Joachim E Wildberger, Harry JGM Crijns, Sebastiaan CAM Bekkers

**Affiliations:** 1 Department of Cardiology, Maastricht University Medical Center, Maastricht, The Netherlands; 2 Radiology, Maastricht University Medical Center, Maastricht, The Netherlands; 3 Cardiovascular Research Institute Maastricht, Maastricht University Medical Center, Maastricht, The Netherlands; 4 Epidemiology, Maastricht University Medical Center, Maastricht, The Netherlands; 5 Department of Cardiology, Maastricht University Medical Center, P. Debyelaan 25, P.O. Box 5800, Maastricht, 6202 AZ, The Netherlands

**Keywords:** Echocardiography, Cardiovascular magnetic resonance, Myocardial infarction

## Abstract

**Background:**

Although echocardiography is used as a first line imaging modality, its accuracy to detect acute and chronic myocardial infarction (MI) in relation to infarct characteristics as assessed with late gadolinium enhancement cardiovascular magnetic resonance (LGE-CMR) is not well described.

**Methods:**

One-hundred-forty-one echocardiograms performed in 88 first acute ST-elevation MI (STEMI) patients, 2 (IQR1-4) days (n = 61) and 102 (IQR92-112) days post-MI (n = 80), were pooled with echocardiograms of 36 healthy controls. 61 acute and 80 chronic echocardiograms were available for analysis (53 patients had both acute and chronic echocardiograms). Two experienced echocardiographers, blinded to clinical and CMR data, randomly evaluated all 177 echocardiograms for segmental wall motion abnormalities (SWMA). This was compared with LGE-CMR determined infarct characteristics, performed 104 ± 11 days post-MI. Enhancement on LGE-CMR matched the infarct-related artery territory in all patients (LAD 31%, LCx 12% and RCA 57%).

**Results:**

The sensitivity of echocardiography to detect acute MI was 78.7% and 61.3% for chronic MI; specificity was 80.6%. Undetected MI were smaller, less transmural, and less extensive (6% [IQR3-12] vs. 15% [IQR9-24], 50 ± 14% vs. 61 ± 15%, 7 ± 3 vs. 9 ± 3 segments, *p* < 0.001 for all) and associated with higher left ventricular ejection fraction (LVEF) and non-anterior location as compared to detected MI (58 ± 5% vs. 46 ± 7%, *p* < 0.001 and 82% vs. 63%, *p* = 0.03). After multivariate analysis, LVEF and infarct size were the strongest independent predictors of detecting chronic MI (OR 0.78 [95%CI 0.68-0.88], *p* < 0.001 and OR 1.22 [95%CI0.99-1.51], *p* = 0.06, respectively). Increasing infarct transmurality was associated with increasing SWMA (*p* < 0.001).

**Conclusions:**

In patients presenting with STEMI, and thus a high likelihood of SWMA, the sensitivity of echocardiography to detect SWMA was higher in the acute than the chronic phase. Undetected MI were smaller, less extensive and less transmural, and associated with non-anterior localization and higher LVEF. Further work is needed to assess the diagnostic accuracy in patients with non-STEMI.

## Background

Myocardial infarction (MI) is a major cause of death and disability worldwide [1]. Accurate diagnosis is important, since it directs clinical management and affects prognosis. Despite the development of cardiac specific biomarkers (i.e. troponins) that increase several hours after the onset of myocardial ischemia, early diagnosis can still be difficult and MI may remain undetected [2]. Non-invasive imaging modalities can improve the diagnosis of MI, due to their ability to detect segmental wall motion abnormalities (SWMA) as a result of myocardial ischemia [3].

Two-dimensional echocardiography has many advantages as a first line, bed-side, real-time imaging modality because it is inexpensive, and rapidly and widely available. Although echocardiography is subsidiary to the electrocardiogram (ECG) in hemodynamically stable patients presenting with ST-segment elevation myocardial infarction (STEMI), its role may be more important in patients with a non-diagnostic ECG. Echocardiography is generally agreed to be very accurate, but the sensitivity to detect acute MI varies widely [4]. Sensitivities of up to 100% have been reported in small studies that investigated patients with previous MI, Q-wave MI and included patients with good image quality [5-8]. However, the sensitivity ranged between 60-70% when predominantly non-Q-wave MI and patients with less optimal image quality echocardiograms were included [9,10]. Most studies have focused on the echocardiographic detection of acute MI, and less is known about its usefulness in chronic MI [11,12]. Chronic MI may remain undetected more frequently, because of the disappearance of SWMA after several weeks [13].

Animal studies have shown that the extent of echocardiographic SWMA is related to infarct size and transmurality [14]. This is also suggested by early clinical studies, but less accurate measures of infarct size were used, such as peak enzyme release and the presence or absence of Q-waves [7,9,10]. The purpose of this study was to investigate the diagnostic accuracy of echocardiography to detect SWMA in the acute and chronic phase in a well described homogeneous population initially presenting with STEMI and consequently a high likelihood of SWMA. Additionally, we investigated the relation of SWMA with underlying infarct characteristics as assessed with late gadolinium enhancement cardiovascular magnetic resonance (LGE-CMR).

## Methods

### Study population

The current study is a retrospective subanalysis of patients admitted with a first ST-elevation MI (STEMI). Patients were consecutively and prospectively enrolled between August 2006 and March 2008. The purpose of the main study was to investigate infarct characteristics at baseline and follow-up using LGE-CMR [15]. For this subanalysis, only patients were studied who had echocardiograms during admission (acute), follow-up (chronic), or both available and in whom LGE-CMR was performed. All patients were referred for urgent coronary angiography and primary percutaneous coronary intervention (PCI) of the infarct related artery (IRA). The definition of STEMI was based on a consensus document that includes an appropriate rise and fall in cardiac biomarkers and electrocardiographic (ECG) changes indicative of new ischemia [16]. Patients ≤18 years, with regular contraindications for CMR, and those with left bundle branch block were excluded. To represent daily clinical practice, patients were not excluded because of poor echocardiographic image quality. In total, 88 patients with 141 echocardiograms were studied (27 acute and 8 chronic echocardiograms were not available). In 53 patients, both acute and chronic echocardiograms were available. During the study period, an arbitrary number of 36 consecutive subjects were extracted from a hospital database to serve as a control group and determine specificity. These subjects were either healthy volunteers or analyzed for various reasons. All were finally diagnosed without cardiac disease and none had evidence of myocardial scar (on LGE-CMR) or significant coronary artery disease (normal invasive coronary angiography, coronary computed tomography, or exercise treadmill test). Maastricht University Medical Center is a high volume center for cardiac ultrasound (13.000 transthoracic and 1000 transoesophageal examinations per year). Informed consent was obtained in all patients and Review Board of Maastricht University Medical Center approved the study (approval number MEC 05-199).

### Echocardiography

Transthoracic echocardiography was performed at a median of 2 (interquartile range [IQR] 1–4) days (acute) and 102 (IQR 92–112) days (chronic) after admission. Echocardiography was performed according to the American Society of Echocardiography guidelines using a commercially available ultrasound system (Sonos 5500 systems with S3 transducers or iE33 systems with S5-1 transducers, Philips Medical Systems, Best, The Netherlands) [17]. All images were acquired in supine or left lateral decubitus position and recorded as ECG-gated digital loops and stored for off-line analysis. Standard parasternal short and long axis views and apical two-, four-, and five-chamber views were obtained. No myocardial contrast enhanced technique was used.

All 177 echocardiographic studies (patients and controls) were analyzed independently and in random order by two board-certified cardiologists (S.S. and S.B.) with >10 years of experience in echocardiography, who were blinded to patient, clinical and CMR data. Discrepancies were resolved in consensus after review by a third expert (E.C.). Left ventricular ejection fraction (LVEF) was estimated visually. Regional wall motion was assessed visually according to the AHA 17-segment model on a four-point scale (0 = normal, 1 = hypokinesia, 2 = akinesia, and 3 = dyskinesia) [18]. Since segments could be visualized in more than one view, a final consensus score was assigned by combining all views. Finally, the presence or absence of MI was assessed (defined as SWMA-score ≥1 in ≥1 segment, with or without wall thinning). Image quality was scored on a three-point scale based on the number of interpretable segments (i.e. 0 = poor, if >1 segment was not interpretable in any view; 1 = average, if all segments were interpretable but not in all views; and 2 = excellent, if all views were interpretable in all views). MI localization was classified as anterior (left anterior descending [LAD] artery territory) or non-anterior (left circumflex [LCx] or right coronary artery [RCA] territory). Only when the localization of SWMA on echocardiography and infarct on LGE-CMR matched, MI was defined as being correctly detected by echocardiography.

### Cardiovascular magnetic resonance

Patients underwent CMR at a mean of 5 ± 2 days (acute) and 104 ± 11 days (chronic) after admission. CMR was performed for research purposes (i.e. not clinically ordered scans), and scan results were not used to guide clinical decision-making. Images were acquired with a 1.5 Tesla system (Philips Intera, Philips Medical Systems, Best, The Netherlands) equipped with a cardiac software package and five-element phased array surface coil. Although our CMR protocol included cine (steady-state free precession) and T2-weighted (black-blood turbo spin echo with fat suppression) imaging, only the LGE-CMR images were used for the purpose of this study. LGE-CMR images were acquired 10 minutes after administration of 0.2mmol/kg body weight Gadolinium diethylenetriaminepentaacetic acid (Gd-DTPA, Magnevist®, Bayer-Schering, Germany), using a breathhold three-dimensional inversion-recovery gradient echo technique (acquired/reconstructed slice thickness 12/6mm, average TR/TE 3.9/2.4ms, multi-shot [50 profiles/shot] segmented partial echo readout, flip angle 15^0^, FOV 400mm, matrix 256x256) in the short axis, two-chamber and four-chamber views [15]. Inversion delay time was set to null signal from normal myocardium (typically 200–280ms).

CMR images were analyzed independently by two observers, experienced in reading CMR and blinded to clinical and echocardiographic data, using commercially available software (CAAS MRV 3.0, Pie Medical Imaging, Maastricht, The Netherlands). The endocardial and epicardial borders were manually traced on the LGE-CMR short axis images, excluding the papillary muscles, to determine infarct size and transmurality. Infarct size and transmurality were measured by manually tracing enhanced areas (including areas of microvascular obstruction) and expressed as a percentage of LV mass and segmental LV wall thickness, respectively. CMR and echocardiographic images were analyzed on separate occasions.

### Statistical analysis

Continuous variables with normally distributed data are expressed as mean ± standard deviation (SD), otherwise as median with IQR. Categorical data are expressed as frequencies with percentages. The inter- and intraobserver agreement between the two readers was analyzed by using Cohen’s kappa (κ) coefficient. Differences in categorical data were evaluated using a Chi-square or Fisher’s exact test. For continuous data, the independent *t*-test was used for normally distributed data and the Mann–Whitney U test when not normally distributed.

Validity of echocardiography for the diagnosis of acute and chronic MI was evaluated by calculation of sensitivity and specificity with corresponding 95% confidence intervals (CI). LGE-CMR was used as reference standard. Differences in sensitivity between acute and chronic MI were tested using a test for paired proportions.

Univariate binary logistic regression was performed to explore the effect of different infarct characteristics on accurate detection of MI by echocardiography. The dependent variable in this analysis was whether or not MI was detected by echocardiography (detected versus undetected). Independent variables associated with a *p*-value <0.05 were selected for inclusion in a multivariate binary logistic regression model in order to evaluate the independent effects of specific infarct characteristics. One variable per every 7–10 events were considered acceptable to be included into the multivariate model, where an event is defined as the outcome that is the least frequent [19].

SPSS version 17.0 (SPSS Inc., Chicago, Illinois) was used for all statistical analyses. A two-tailed value of *p* < 0.05 was considered statistically significant.

## Results

### Baseline characteristics

Patients with MI were older and more often active or ex-smokers than healthy controls (59 ± 11 vs. 43 ± 12 years and 86% vs. 11%, respectively, *p* < 0.001 for both, Table 1). In patients, post-PCI thrombolysis in myocardial infarction (TIMI 3) flow was established in 88%. The infarct-related artery (IRA) was the LAD in 31%, LCx in 12% and RCA in 57% of patients, and approximately half had single vessel disease (51%). In all patients, enhancement was visible on LGE-CMR images matching the territory of the IRA. Infarct size and infarct transmurality were 11% (IQR5-19) and 57 ± 16%, respectively. Infarct size was smaller and LVEF higher in patients with non-anterior MI than in patients with anterior MI (10% [IQR5-15] vs. 23% [IQR13-28] and 52 ± 8% vs. 45 ± 10%, respectively, *p* < 0.001 for both).

**Table 1:** Baseline characteristics

	**Patients (N** **=** **88)**	**Controls (N** **=** **36)**	***p-*value**
Age, y	59 ± 11	43 ± 12	<**0**.**001**
Male (%)	65 (74)	20 (56)	0.06
Diabetes mellitus (%)	6 (7)	1 (3)	0.68
Smoking (%)	76 (86)	4 (11)	<**0**.**001**
Hypertension (%)	34 (39)	7 (19)	0.80
Hypercholesterolemia (%)	25 (28)	4 (11)	0.18
Positive family history (%)	41 (47)	14 (39)	0.84
**Coronary Angiography**			
Infarct related artery (%)			
LAD	27 (31)	-	
LCx	11 (12)	-	
RCA	50 (57)	-	
Number of diseased vessels (%)			
1	45 (51)	-	
≥2	43 (49)	-	
TIMI 3 (%)			
Pre-PCI	8 (9)	-	
Post-PCI	77 (88)	-	
**Echocardiography**			
Days post MI			
Acute	2 (1–4)	-	
Chronic	102 (92–112)	-	
Image quality (%)			0.87
Excellent	42 (30)	9 (25)	
Average	87 (62)	24 (67)	
Poor	12 (8)	3 (8)	
**CMR**			
Days post MI			
Acute	5 ± 2		
Chronic	104 ± 11		
Days between chronic echo and CMR	0 (0–4)	38 (13–76)	
Infarct size, % of LV	11 (5–19)	-	
Infarct transmurality, %	57 ± 16	-	
Number of infarcted segments	8 ± 3	-	

Values are presented as mean ± standard deviation, median and interquartile range or proportions (%) when appropriate. LAD = left anterior descending artery; LCx = left circumflex artery; RCA = right coronary artery; TIMI = thrombolysis in myocardial infarction PCI = percutaneous coronary intervention; MI = myocardial infarction; CMR = cardiovascular magnetic resonance; LVEF = left ventricular ejection fraction.

### Intraobserver and interobserver agreement

Analysis of intraobserver variability of echocardiographic assessment showed an agreement of 80% (κ = 0.58) and 85% (κ = 0.63) for observer 1 and 2, respectively. Analysis of the interobserver variability showed an agreement of 85% (κ = 0.70). The interobserver agreement for measuring infarct size on LGE-CMR images was excellent (κ = 0.90).

### Diagnostic performance of echocardiography

The diagnostic performance of echocardiography to detect MI is shown in Table 2. Overall, MI was detected by echocardiography in 97 out of 141 studies, resulting in an overall sensitivity of 68.8%. Forty-eight out of the 61 patients with acute MI, and 49 out of the 80 patients with chronic MI were detected, resulting in a sensitivity of 78.7% and 61.3%, respectively. In the 53 patients in whom both acute and chronic echocardiograms were available for analysis, sensitivity for acute and chronic MI were 75.4% (40/53) and 67.9% (36/53). This was not significantly different as compared to the total group (*p =* 0.84 and *p* = 0.06, respectively). The sensitivity to detect LCx-related MI was somewhat higher than RCA-related MI, but this did not reach statistical significance (70.0% vs. 61.0%, respectively, *p* = 0.46)

**Table 2:** Diagnostic performance of echocardiography to detect myocardial infarction

	**N**	**Sensitivity 95% CI**	**Specificity 95% CI**	**PPV 95% CI**	**NPV 95% CI**
**Overall**	141	68.8% (64.9-71.4)	80.6% (65.3-90.9)	93.3% (88.0-96.8)	39.7% (32.2-44.8)
**Acute MI**	61	78.7% (70.8-84.4)	80.6% (67.1-90.2)	87.3% (78.5-93.6)	69.0% (57.6-77.3)
**Chronic MI**	80	61.3% (54.8-65.8)	80.6% (66.1-90.7)	87.5% (78.2-94.0)	48.3% (39.7-54.4)

CI = confidence interval; PPV = positive predictive value; NPV = negative predictive value; MI = myocardial infarction.

Overall, the localization of observed SWMA did not match the localization of infarction on LGE-CMR in 4 out of 141 studies (2.8%) and more frequently so in chronic than in acute MI (3.8% vs. 1.6%). In healthy controls, 7 out of 36 studies were incorrectly classified as MI, resulting in a specificity of 80.6%. All false positive assessments concerned the basal inferior or basal inferolateral segments.

### Characteristics of detected and undetected myocardial infarction

Echocardiographically undetected MI were more often non-anteriorly located in comparison to detected MI (82% vs. 63%, *p* = 0.03, Table 3). The prevalence of multivessel disease did not differ between patients with undetected and detected MI (*p* = 0.85). Overall, undetected MI were smaller, less transmural, involved less segments, and were associated with higher LVEF as compared to detected MI (6% [IQR3-12] vs. 15% [IQR9-24], 50 ± 14% vs. 61 ± 15%, 7 ± 3 vs. 9 ± 3 segments, and 58 ± 5% vs. 46 ± 7, respectively, *p* < 0.001 for all). Image quality was not different between patients with undetected and detected MI. Similar associations with infarct characteristics were found in the 53 patients who had both acute and chronic echocardiograms.

**Table 3:** Comparison of characteristics between detected and undetected myocardial infarctions

	**Detected (N** **=** **97)**	**Undetected (N** **=** **44)**	***p-*value**
Age, y	60 ± 12	59 ± 10	0.81
Male (%)	74 (76)	33 (75)	0.87
Infarct localization (%)			**0**.**03**
Anterior	36 (37)	8 (18)	
Non-anterior	61 (63)	36 (82)	
Number of diseased vessels			0.85
1	49 (51)	23 (52)	
≥2	48 (49)	21 (48)	
**Echocardiography**			
LVEF (%)			
Acute	44 ± 7	58 ± 7	<**0**.**001**
Chronic	48 ± 7	59 ± 5	<**0**.**001**
Days post MI			
Acute	2.0 (1.0-3.0)	4.0 (3.0-6.5)	**0**.**001**
Chronic	101 (91–113)	104 (97–111)	0.50
Image quality (%)			0.37
Excellent	26 (27)	16 (36)	
Average	61 (63)	26 (59)	
Poor	10 (10)	2 (5)	
**CMR**			
Infarct size, % of LV			
Acute	16 (10–25)	6 (3–13)	**0**.**002**
Chronic	15 (8–23)	6 (3–11)	<**0**.**001**
Infarct transmurality, %			
Acute	62 ± 14	54 ± 17	0.07
Chronic	61 ± 16	48 ± 13	<**0**.**001**
Number of infarcted segments			
Acute	9 ± 3	7 ± 3	**0**.**02**
Chronic	9 ± 3	7 ± 3	**0**.**005**

Values are presented as mean ± standard deviation, median and interquartile range or proportions (%) when appropriate; MI = myocardial infarction; CMR = cardiovascular magnetic resonance; LVEF = left ventricular ejection fraction.

#### Acute versus chronic myocardial infarction

In both acute and chronic MI, LVEF was significantly higher in undetected than in detected MI (58 ± 7% vs. 44 ± 7% and 59 ± 5% vs. 48 ± 7%, respectively, *p* < 0.001 for both, Table 3) and the probability of detecting MI increased as LVEF decreased (b = -0.30 and b = -0.27, respectively, *p* < 0.001 for both, Table 4).

**Table 4:** Univariate and multivariate logistic regression analysis for the prediction of the echocardiographic detection of myocardial infarction

	**Univariate**			**Multivariate**		
	**Coefficient (B)**	**OR (95% CI)**	***p-*value**	**Coefficient (B)**	**OR (95% CI)**	***p-*value**
**Acute MI**						
Infarct localization						
*Anterior vs. non-anterior*	2.06	7.86 (0.94-65.5)	0.06			
Image quality						
*Poor vs. excellent*	0.49	1.64 (0.14-19.4)	0.70			
*Average vs. excellent*	-0.06	0.94 (0.22-4.11)	0.93			
Time point echocardiography	-0.32	0.72 (0.56-0.94)	**0**.**014**			
LVEF	-0.30	0.74 (0.63-0.87)	<**0**.**001**			
Infarct size	0.16	1.17 (1.05-1.31)	**0**.**006**			
Infarct transmurality	0.04	1.04 (1.00-1.09)	0.08			
Number of infarcted segments	0.28	1.33 (1.04-1.71)	**0**.**026**			
**Chronic MI**						
Infarct localization						
*Anterior vs. non-anterior*	0.60	1.82 (0.65-5.09)	0.25			
Image quality						
*Poor vs. excellent*	1.24	3.47 (0.34-35.1)	0.29			
*Average vs. excellent*	0.43	1.53 (0.59-3.96)	0.38			
Tme point echocardiography	-0.02	0.98 (0.94-1.01)	0.18			
LVEF	-0.27	0.76 (0.68-0.86)	<**0**.**001**	-0.25	0.78 (0.68-0.88)	<**0**.**001**
Infarct size	0.16	1.17 (1.08-1.28)	<**0**.**001**	0.20	1.22 (0.99-1.51)	0.06
Infarct transmurality	0.06	1.06 (1.02-1.10)	**0**.**001**	-0.02	0.98 (0.92-1.04)	0.46
Number of infarcted segments	0.25	1.28 (1.07-1.53)	**0**.**010**	-0.11	0.89 (0.61-1.32)	0.58

OR = odds ratio; CI = confidence interval; MI = myocardial infarction; LVEF = left ventricular ejection fraction.

In patients with undetected acute MI, echocardiography was performed later after admission than in patients in whom acute MI was detected (4.0 [IQR3.0-6.5] vs. 2.0 [IQR1.0-3.0] days, *p* = 0.001). Accordingly, the probability of detecting an acute MI was inversely related to the delay time between admission and performance of echocardiography (b = -0.32, *p* = 0.014, Table 4). This association was not found in chronic MI.

**Figure 1:**
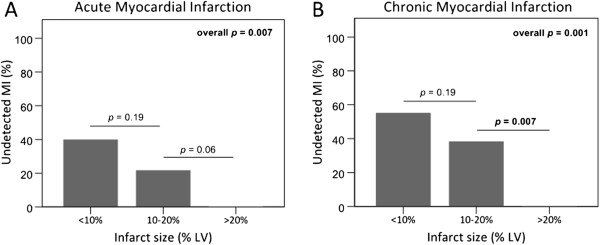
**Undetected myocardial infarction in relation to infarct size.** The prevalence of undetected myocardial infarction decreased with increasing infarct size in acute (**A**) and chronic (**B**) myocardial infarction.

**Figure 2:**
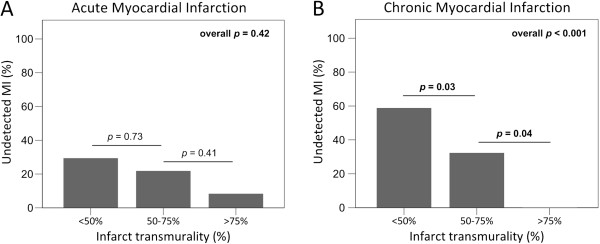
**Undetected myocardial infarction in relation to infarct transmurality.** The prevalence of undetected myocardial infarction decreased with increasing infarct transmurality in acute (**A**) and chronic myocardial infarction (**B**), and was significant in chronic myocardial infarction only.

The probability of accurately detecting acute and chronic MI increased with increasing infarct size (b = 0.16 for both, *p* < 0.001, respectively [Table 4, Figure 1A and 1B]), increasing infarct transmurality (b = 0.04, *p* = 0.08 and b = 0.06, *p* = 0.001, respectively, [Table 4, Figure 2A and 2B]), and increasing number of infarcted segments (b = 0.28, *p* < 0.05 and b = 0.25, *p* < 0.01, respectively, [Table 4]). All >75% transmural chronic MI were detected (Figure 2B), while one >75% transmural, small acute MI (infarct size 3%) remained undetected (Figure 2A).

Univariate regression analysis revealed that the detection of acute MI was significantly associated with the time point of echocardiography after admission, LVEF, infarct size and segmental extent of infarction (Table 4). Due to the limited number of undetected acute MI, a multivariate logistic regression analysis to evaluate the independent effects of specific infarct characteristics was considered feasible only with respect to chronic MI. The results of a multivariate model, where LVEF, infarct size, infarct transmurality and the number of infarcted segments were entered as independent variables, indicated that only LVEF and infarct size were strongly and independently associated with undetected chronic MI (b = -0.25 and b = 0.20, *p* < 0.001 and *p* = 0.06, respectively). Similarly, multivariate regression analysis in the 53 patients with serial echocardiograms resulted in similar associations for both LVEF and infarct size (b = -0.25 and b = 0.08).

An example of a patient with undetected chronic MI is available online (Additional files 1 and 2).

### Segmental analysis

**Figure 3:**
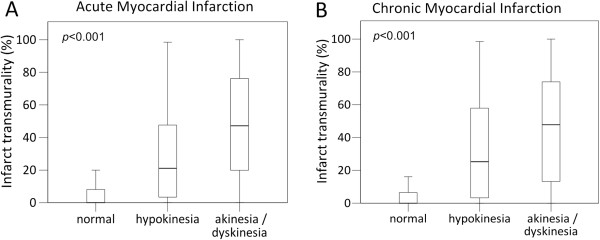
**Relationship of echocardiographic segmental wall motion abnormality and infarct transmurality.** Box plots illustrating the positive relationship of echocardiographic segmental wall motion abnormality with infarct transmurality in acute (**A**) and chronic (**B**) myocardial infarction (*p* < 0.001 for both).

A total of 2370 segments (91%) were evaluated for SWMA in relation to infarct transmurality by LGE-CMR. A significant positive relationship was found between the severity of SWMA and increasing infarct transmurality (*p* < 0.001 for both acute and chronic MI, Figure 3A and B). XIn acute and chronic MI, median values of infarct transmurality were 0% (IQR0-8%) and 0% (IQR0-7%) in normokinetic segments, 21% (IQR3-48) and 25% (IQR3-58) in hypokinetic segments and 47% (IQR20-77) and 48% (IQR10-75) in akinetic segments, respectively.

## Discussion

We found that the overall sensitivity of regular transthoracic 2D-echocardiography to detect MI is modest. For acute, several days-old MI (first STEMI), its sensitivity was moderate and even lower for chronic, 3 months-old MI. Undetected MI were smaller, less extensive and less transmural and associated with non-anterior localization and higher LVEF.

Accurate detection of MI is important but can be challenging, especially when symptoms are atypical and electrocardiographic changes are non-specific or absent (non-STEMI). Undetected MI account for at least one-fifth of all infarctions and carry a prognosis as poor as detected MI [20]. Detecting new loss of myocardium or new SWMA is currently a key part of the universal definition of MI, and imaging modalities have been added to cardiac biomarker testing [16], and given a class 1 recommendation in acute coronary syndrome guidelines [21].

### Acute myocardial infarction

Although a number of studies are being referred to as having shown a high diagnostic accuracy of echocardiography to detect acute MI, several of them were not designed as such or used suboptimal methods to determine infarct size and localization [4]. Heger et al. investigated patients with Q-wave MI and found SWMA in the entire study population. Q-wave MI are predominantly related to infarct size rather than infarct transmurality [22]. The high incidence of cardiogenic shock, heart failure and deaths, confirms that predominantly patients with large MI were studied, positively affecting sensitivity. Of note, 7 out of 44 echocardiograms could not be evaluated and the true sensitivity is therefore lower [5]. In another study of 90 patients with a first acute MI, SWMA correlated well with the location of MI on the ECG. All patients had a Q-wave MI, and thus presumably larger MI. Again, only 73% of echocardiograms could be evaluated. The authors mention the moderate success in obtaining adequate images in the acute setting as an important limitation of echocardiography [6]. Gibson et al. reported that SWMA were detected in all of 75 patients consecutively admitted with acute MI. However, one-third of patients had a previous MI of whom two-thirds showed SWMA outside the electrocardiographic infarct zone. In this setting, the sensitivity to detect acute MI was overestimated since echocardiography might detect a coexistent large chronic MI while missing a smaller acute MI. In addition, 16 (21%) patients developed cardiogenic shock of whom 14 (88%) died, again suggesting that mainly patients with large MI were studied [7]. In another study of 43 patients with chest pain and non-diagnostic electrocardiograms, 13 developed a MI of which 12 (92%) were detected by echocardiography. However, only patients with good image quality were analyzed and no information was provided on the agreement between SWMA and electrocardiographic location of infarction [8]. In contrast, two other studies investigating patients with acute non-Q-wave and no previous MI found sensitivities of 66% and 83%, comparable to our findings [9,10].

The wide variation in sensitivity can be explained by the fact that many studies were small, used different in- and exclusion criteria, or used no or less appropriate reference standards to compare SWMA with. To our knowledge, our study is the largest thus far, specifically addressing the diagnostic accuracy of echocardiography to detect SWMA in comparison to LGE-CMR in a homogenous population of patients with STEMI. We used LGE-CMR as the clinical reference standard to correlate SWMA with. In our study, correct echocardiographic identification of MI was defined when the location of echocardiographic SWMA matched the infarct region on LGE-CMR images, thereby preventing false positives (i.e. classification of an infarct by echocardiography if SWMA were observed in a remote region [4 out of 141 echocardiograms (2.8%)]).

### Chronic myocardial infarction

While most studies have focused on the diagnostic accuracy of echocardiography to detect acute MI, less is known about its usefulness in chronic MI. We found that the sensitivity of echocardiography to detect chronic MI was even lower than for acute MI. This was true in the population as a whole as well as in the cohort of 53 patients with serial echocardiograms. This lower sensitivity can be explained by improvement of contractility of initially “stunned” myocardium several months after successful revascularization [23]. It also has important clinical implications, because not infrequently, cardiologists are confronted with patients presenting with coincident pathological Q-waves and a normal echocardiogram. In these cases, either the ECG can be false-positive or the echocardiogram false-negative for the diagnosis of chronic MI. To prevent that appropriate therapy is withheld in these patients, LGE-CMR can be applied to definitively rule in or rule out chronic MI. Advanced echocardiographic techniques such as speckle tracking may also play a role to improve diagnostic accuracy.

### Relationship with infarct characteristics and LVEF

MI was more frequently undetected in patients with higher LVEF, smaller, less transmural and less extensive infarctions and when non-anteriorly located. For undetected acute MI the relationship with infarct transmurality was less strong than for chronic undetected MI, most likely as a result of “stunning” (Figure 2). The relationship between infarction and contractile function is complex. CMR studies have shown that contractility improves over time in dysfunctional myocardium with <25% transmural infarction [13], and that the likelihood of functional recovery decreases with increasing infarct transmurality [24]. The relation of contractile function with infarct transmurality also applies for chronic MI. Blinded observers were unable to detect SWMA in 37% of infarcted segments, of which 84% were <50% transmurally infarcted [25]. In our study, all >75% transmural chronic MI were correctly identified.

We also found a significant relationship of the severity of echocardiographic SWMA with increasing infarct transmurality (Figure 3), confirming the results of a previous study in 15 autopsied patients in whom ante-mortem echocardiograms were compared with pathology. In that study, all transmurally infarcted segments were akinetic or dyskinetic, and 71% of subendocardially infarcted segments were either hypokinetic or normal [26]. Regardless whether echocardiography or CMR was used, studies have repeatedly shown the relationship of contractility with infarct characteristics. Therefore, MI may remain undetected when the diagnosis is only based on the presence or absence of SWMA.

Echocardiography is a very useful imaging modality in patients with acute chest pain because it can also detect life threatening conditions other than acute MI, such as acute aortic dissection, cardiac tamponade, or pulmonary embolism. It is not our intention to bring echocardiography into disrepute, but rather to call for a cautious approach when it is used as a final test to rule out MI.

### Limitations

Although we did not use contrast echocardiography to improve endocardial border definition [27], we believe this would not have changed our results. First, image quality was not an important predictor for the detection of both acute and chronic MI in our study. Second, a small, subendocardial infarction would still appear normal and therefore remain undetected due to normal contracting neighboring segments (‘inverse tethering’) [25]. Echocardiographic strain imaging may have increased the sensitivity to detect MI, as this technique was previously shown to correlate well with the presence and extent of scar on LGE-CMR [28]. However, accurate strain analysis necessitates additional software and off-line analysis that is less practical in an emergency setting. Whether it may improve the diagnostic accuracy to detect a chronic MI needs further investigation. We cannot exclude that misregistration between echocardiographic and LGE-CMR images affected our results, although segments were visualized in multiple views in the majority of patients.

We investigated a homogenous study population, albeit with a modest sample size. Echocardiograms were not available in all patients, and this may have affected our results. At the time of the first echocardiogram (median 2 days, IQR1-4), SWMA might have resolved in some patients, lowering sensitivity. However, the process of myocardial stunning after acute MI generally lasts days to weeks [29]. Although automatic quantification of infarct size and transmurality might have improved accuracy, manual adjustment of contours often remains necessary. Furthermore, no consensus exists on what signal intensity thresholds should be used.

## Conclusions

The sensitivity of regular echocardiography to detect SWMA in patients initially presenting with STEMI is moderate in the acute phase and even lower in the chronic phase. Undetected MI were smaller, less extensive and less transmural, and associated with non-anterior localization and higher LVEF. Although echocardiography is recommended by current acute coronary syndrome guidelines, excluding MI solely based on wall motion should be done cautiously. When clinical suspicion remains high and all other diagnostic tests are inconclusive, LGE-CMR can be applied.

## Abbreviations

ECG: Electrocardiogram; IRA: Infarct related artery; LGE-CMR: Late gadolinium enhancement cardiovascular magnetic resonance; LVEF: Left ventricular ejection fraction; MI: Myocardial infarction; PCI: Percutaneous coronary intervention; SWMA: Segmental wall motion abnormalities; STEMI: ST-elevation myocardial infarction

## Competing interests

The authors declare that they have no competing interests.

## Authors’ contributions

All authors have contributed to the conception and design of the study, the interpretation of data, and have critically revised and subsequently approved the manuscript. CJ, SS, EC, MS, ID, PN and SB performed collection and analysis of the data.

## Supplementary Material

Additional file 1 **Undetected chronic inferior myocardial infarction.** Corresponding echocardiographic (left) and late gadolinium enhancement cardiovascular magnetic resonance (right) short axis views at mid-ventricular level. Click here for file

Additional file 2 **Undetected chronic inferior myocardial infarction.** Corresponding echocardiographic (left) and late gadolinium enhancement cardiovascular magnetic resonance (right) two-chamber views. Click here for file
